# Experimental Investigation on the Flexural Performance of CFRP-Reinforced Timber Composite Beams

**DOI:** 10.3390/ma19061196

**Published:** 2026-03-18

**Authors:** Hao Zhang, Yan Cao, Hai Fang, Honglei Xie, Chen Chen

**Affiliations:** 1 College of Civil Engineering, Nanjing Tech University, Nanjing 211816, China; zhanghao@njtech.edu.cn (H.Z.); fanghainjut@163.com (H.F.); 2 College of Materials Science and Engineering, Nanjing Tech University, Nanjing 211816, China; caoyan@njtech.edu.cn; 3 Department of Civil Engineering and Architecture, Anhui University of Technology, Maanshan 243099, China; hongleixie94@ahut.edu.cn

**Keywords:** CFRP, FRP–timber composite beam, flexural behavior, failure mode

## Abstract

The development of lightweight, high-strength structural systems is a persistent pursuit in modern civil engineering. This paper presents an experimental study on a novel hybrid beam concept in which a sawn timber core is fully bonded with an externally applied Carbon Fiber-Reinforced Polymer (CFRP) laminate, fabricated through a controlled hand lay-up process. The design seeks to exploit the complementary characteristics of the two materials: timber provides compressive resistance and serves as a permanent formwork, while the CFRP carries tensile stresses with high efficiency. Fourteen hybrid beams, with variations in the number of longitudinal CFRP layers (one, two or, three), the presence or absence of longitudinal CFRP layers bonded along the top and bottom surfaces, and the presence or absence of circumferential wrapping in the pure bending region, were tested under four-point bending alongside two solid timber control beams. The results demonstrate that circumferential wrapping is a critical design detail. Wrapped beams consistently failed by tensile rupture of the CFRP—the intended failure mode—and exhibited ultimate moments 15–20% higher than their unwrapped counterparts. Beams with two longitudinal CFRP layers offered the most favorable balance between strength enhancement and material efficiency; adding a third layer shifted the failure mode to crushing of the timber core, indicating a core-limited condition. All hybrid beams showed pronounced linear-elastic behavior up to sudden brittle failure, with performance variability attributable to the inherent inhomogeneity of wood and the sensitivity of the hand lay-up process. The study provides quantitative data and mechanistic insights that support the design and application of bonded CFRP–timber hybrid beams as efficient structural members.

## 1. Introduction

The construction industry, characterized by its conservatism and emphasis on cost-effectiveness, has been slower than other sectors in adopting advanced materials. While materials science has undergone revolutions, construction practice often relies on conventional materials like concrete, steel, and timber. However, growing demands for sustainable, resilient, and high-performance infrastructure are driving the exploration of innovative material solutions. Fiber-Reinforced Polymer (FRP) composites, particularly Carbon FRP (CFRP), offer a compelling suite of properties: high specific strength and stiffness, excellent corrosion and fatigue resistance, electromagnetic neutrality, and tailorable anisotropy [1,2,3,4]. These attributes position CFRPs as promising candidates to address contemporary engineering challenges, especially in applications where weight reduction, durability, and rapid deployment are critical, such as long-span bridges, seismic retrofitting, and modular structures [5,6,7,8,9].

Current civil engineering applications of CFRP are predominantly in the domain of strengthening and rehabilitating existing structures, utilizing externally bonded strips or sheets [10,11,12]. Their use as primary load-bearing elements in new construction remains nascent, often hindered by high material costs, lack of comprehensive design codes, and limited understanding of their long-term behavior in structural systems [13]. A promising strategy to optimize cost-performance and leverage composite advantages is the concept of hybrid members, where CFRP is strategically combined with traditional materials [14,15,16]. In this context, timber, a renewable and lightweight material with favorable compressive strength [17], presents an ideal candidate for a composite core. The CFRP skin can carry tensile stresses efficiently, while the timber core provides stability, shear resistance, and a form for the composite during fabrication [18,19,20,21,22].

Researchers widely use FRP to strengthen timber cores, with CFRP standing out due to its high strength and efficiency. Brol et al. [23] employed CFRP, BFRP, and GFRP to repair and reinforce deteriorated timber, finding that specimens strengthened with CFRP exhibited the highest average bending capacity (a 60.66% increase compared to unreinforced members). Dewey et al. [24] used CFRP and GFRP to repair and reinforce deteriorated timber beams and conducted bending performance tests, revealing that FRP enhanced stiffness by 30% by bridging localized defects and crack openings. Liu et al. [25] investigated the effect of the number of CFRP reinforcement layers on the flexural performance of wood beams and found that the flexural capacity of wood beams with one and two layers of CFRP fabric increased by an average of 14.7% and 23.1%, respectively. Nadir et al. [26] studied the flexural performance of wood beams reinforced with CFRP and GFRP. By applying the same proportion of CFRP and GFRP to the tension side of the beams, the flexural strength increased by 45.86% and 36.91%, respectively, demonstrating the outstanding advantages of CFRP.

Additionally, researchers have proposed various innovative CFRP-reinforced timber composite components. Nabati et al. [27] developed a novel wood–steel–CFRP hybrid beam and tested its bending performance. The study found that the maximum load capacity increased by 4.5 times compared to the reference specimens, and the presence of wood helped restrain buckling. Waqas et al. [28] proposed a novel sandwich composite panel with CFRP reinforcement and various types of wood cores, and found that the optimal configuration ([45/−45] lay-up angle, using carbon fiber-reinforced polymer and a paulownia core) achieved a significant weight reduction to 11.3 tons, nearly half that of an equivalent steel shell. Yang et al. [29] used CFRP bars to enhance the flexural performance of glulam beams. The test results showed that the flexural capacity of the reinforced, prestressed, and prestressed & reinforced (prestressed at the bottom and reinforced at the top) beams significantly increased by 64.8%, 93.3%, and 131%, respectively.

Although the above FRP–timber components are conceptually attractive, there are still significant research gaps. The structural performance of such hybrid CFRP–timber members is highly sensitive to the interfacial bond integrity, fabrication process (which co-determines material and structural properties), and the complex interaction between orthotropic constituents under load [30,31]. The brittle nature of both CFRP and timber raises concerns about failure warning and redundancy.

**Figure 1:**
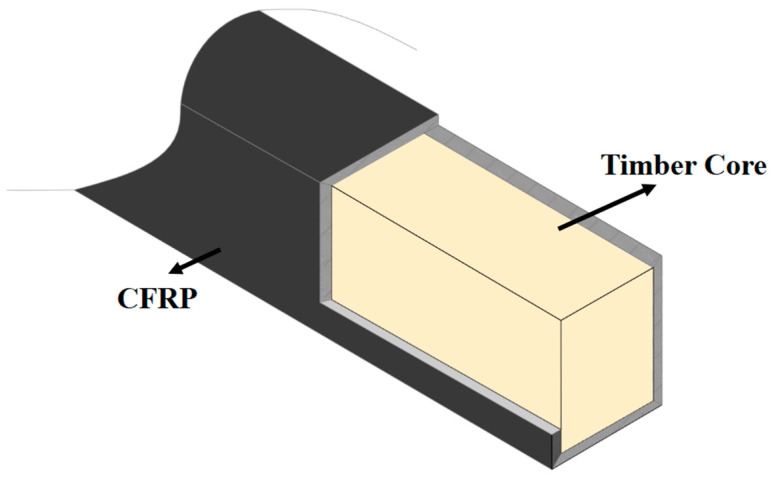
Structural schematic diagram of CFRP reinforced timber core beams.

To address these gaps, this study undertakes a systematic investigation into the flexural behavior of a novel timber-cored CFRP composite beam, as shown in Figure 1. The specific objectives are: (1) to develop a feasible fabrication protocol using the hand lay-up process; (2) to experimentally characterize the failure modes, load–deflection response, and strain development of beams with different CFRP configurations, including the number of CFRP layers, the application of additional longitudinal CFRP layers along the top and bottom surfaces, and the use of transverse CFRP wrapping in the pure bending region; (3) to quantify the CFRP lay-up on flexural performance. This design is primarily based on the consideration that the pure bending region is the critical zone where the beam experiences the maximum bending moment. By concentrating the CFRP reinforcement in this area, the external composite layers can directly and effectively participate in flexural resistance. The findings aim to contribute to the fundamental knowledge base and provide practical guidelines for the design and application of this lightweight, high-strength structural system.

## 2. Experiment Set-Up

### 2.1. Material Properties

Cunninghamia lanceolata timber was used as the core due to its advantages of being lightweight with high strength, high yield, and moderate price. The density of the timber is 0.5 g/cm^3^. Compression tests and three-point bending tests were conducted in accordance with ASTM D143-25 [32] to determine its mechanical properties. Three specimens with dimensions of 200 mm × 50 mm × 50 mm were used for the compression tests, and three specimens with dimensions of 25 mm × 25 mm × 410 mm were used for the bending tests. All specimens were kiln-dried prior to testing to condition the moisture content to 12%, and the actual moisture content was determined and verified as required by ASTM D143-25 [32]. The test results revealed the following characteristic strengths: bending strength of 11 ± 2.3 MPa (mean ± SD, n = 3), longitudinal compressive strength of 10 ± 2.7 MPa, and compressive modulus of elasticity of 9.0 ± 1.0 GPa.

The reinforcement material used was CFW300 unidirectional carbon fiber fabric, which is processed from Japanese-produced T700, 12K carbon fibers, and specialized composite fibers. According to the test results from the Fourth Research Institute of the Nanjing Fiberglass Research and Design Institute, the unidirectional carbon fiber fabric (CFRP) has a nominal tensile strength of 3000 MPa, an elastic modulus of 210 GPa, an ultimate strain of 1.4%, and an areal weight resulting in a ply thickness of approximately 0.167 mm.

The adhesive used for the specimens was Sikadur-330, a two-component epoxy resin specifically designed for bonding CFRP to various substrates, including timber, with an elastic modulus of 4500 MPa and a tensile strength of 30 MPa as provided by the manufacturer (Sika AG, Baar, Switzerland); the adhesive was applied using the hand lay-up process during fabrication.

### 2.2. Specimen Design and Fabrication

The fundamental beam geometry was a 1000 mm long rectangular section of 100 mm × 50 mm, with 10 mm radius fillets on all four longitudinal edges to prevent stress concentration in the CFRP layers. This 10:1 length-to-height ratio was selected to reflect the typical slenderness ratios found in practical structural components such as floor beams and bridge girders, ensuring flexure-dominated behavior rather than shear while also accommodating testing apparatus limitations and maintaining a reasonable scale for observing failure mechanisms.

Three types of CFRP reinforcement schemes were employed in this study: (1) an increased number of CFRP layers, (2) additional longitudinal CFRP layers bonded along the top and bottom surfaces, and (3) transverse CFRP wrapping applied in the pure bending region. Intermittent wrapping, rather than continuous wrapping, was adopted for the transverse reinforcement. This configuration was intentionally designed to simulate the discontinuous reinforcement schemes commonly used in practical retrofitting projects, where limitations in material quantity, construction conditions, or costs often necessitate such an approach.

The experimental matrix comprised 7 series, each containing 2 nominally identical beams. A total of 14 composite beams and 2 solid timber control beams (C_1–2_) were fabricated. The designation of composite beams followed the format Wx-ByTz-Pd, where Wx represented specimens fully wrapped with CFRP layers on all sides, By denoted specimens with additional CFRP reinforcement on the bottom surface, Tz denoted specimens with additional CFRP reinforcement on the top surface, and Pd represented specimens with transverse CFRP wrapping in the pure bending region. The subscripts x, y, z = 1, 2, 3 indicated the number of CFRP layers applied, while d denoted the spacing of the transverse CFRP wraps. If a specimen did not include a particular reinforcement style, the corresponding designation was omitted. For example, W3 represented a specimen fully wrapped with three layers of CFRP on all sides; W1-B1T1-P50 represented a specimen fully wrapped with one layer of CFRP, with one additional CFRP layer on both the top and bottom surfaces, and transverse CFRP wrapping in the pure bending region at a spacing of 50 mm.

**Figure 2:**
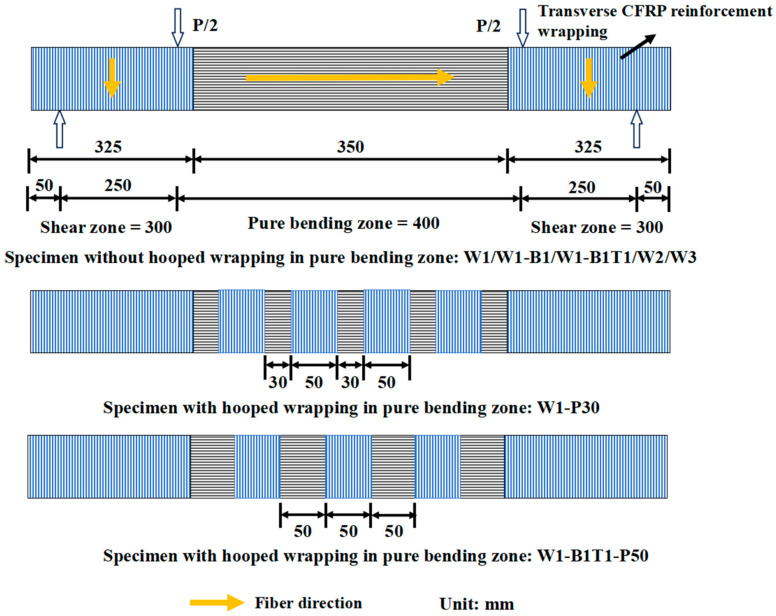
Schematic diagram of CFRP layout.

**Figure 3:**
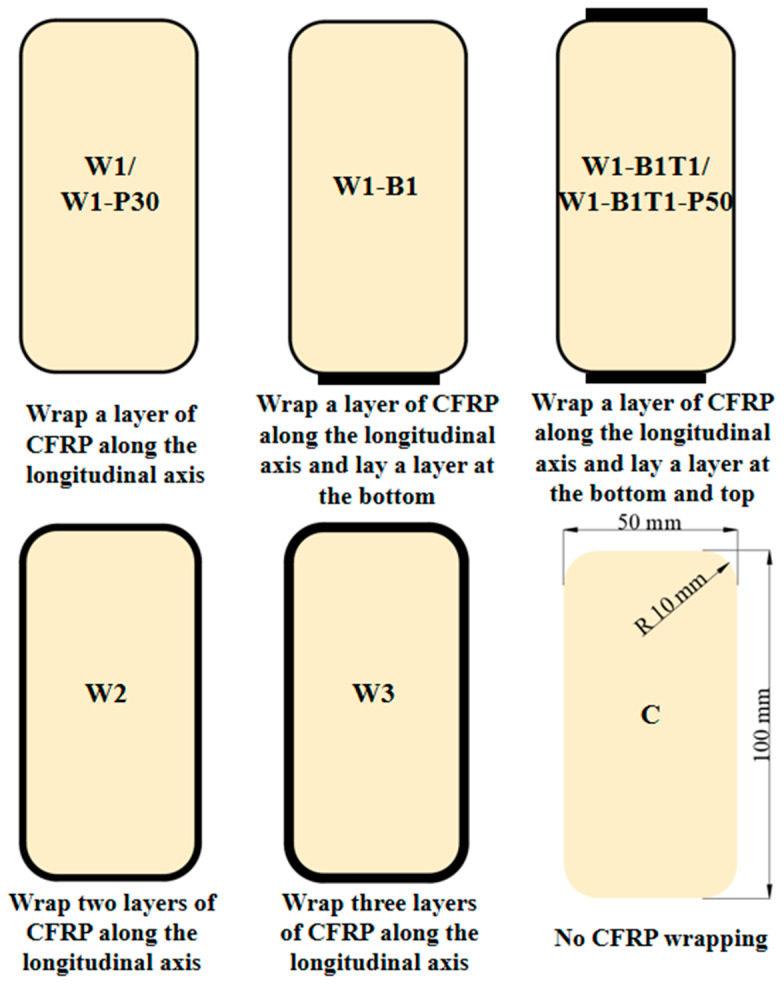
Sectional form of each group of specimens.

Fabrication followed a meticulous hand lay-up procedure: (1) Timber cores were planed, sanded, and cleaned. (2) Epoxy resin was applied to the core surface. (3) Pre-cut CFRP sheets were laid sequentially—first longitudinal layers (butt-jointed), then any additional face strips, and finally circumferential wraps (30 mm lap length). (4) A roller was used to impregnate fibers, remove air voids, and ensure conformity. (5) Beams were cured at ambient temperature for 24 h, followed by post-curing at 70 °C for 4 h, and then slowly cooled to room temperature. The key variable parameters are summarized in Table 1. The CFRP layout of specimens is shown in Figure 2 and Figure 3.

### 2.3. Experimental Set-Up

**Figure 4:**
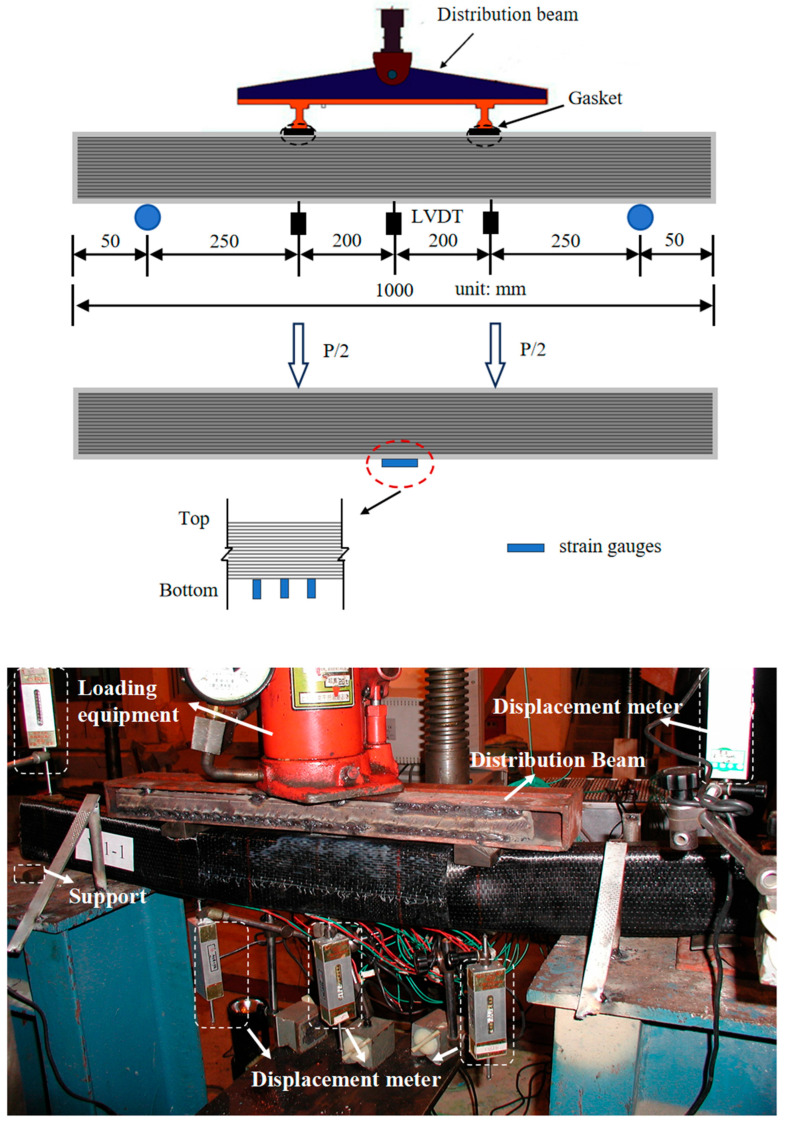
Test set-up and strain gauge arrangement.

Four-point bending tests were conducted over a simply supported span of 900 mm with a constant pure bending region of 400 mm and a shear region of 250 mm (Figure 4). A servo-hydraulic actuator applied load through a spreader beam. The load was measured with a load cell. Deflection at mid-span and supports was monitored using Linear Variable Differential Transformers (LVDTs) produced by Jiangsu Liyang Electronic Instrument Co., Ltd., (Changzhou, China). Strain distributions across the depth at mid-span were captured using electrical resistance strain gauges mounted on the CFRP surface and a mechanical demec gauge for longer gauge lengths. The load was applied in displacement control at a slow rate of 2 mm/min to capture pre-failure behavior.

## 3. Failure Modes

**Figure 5:**
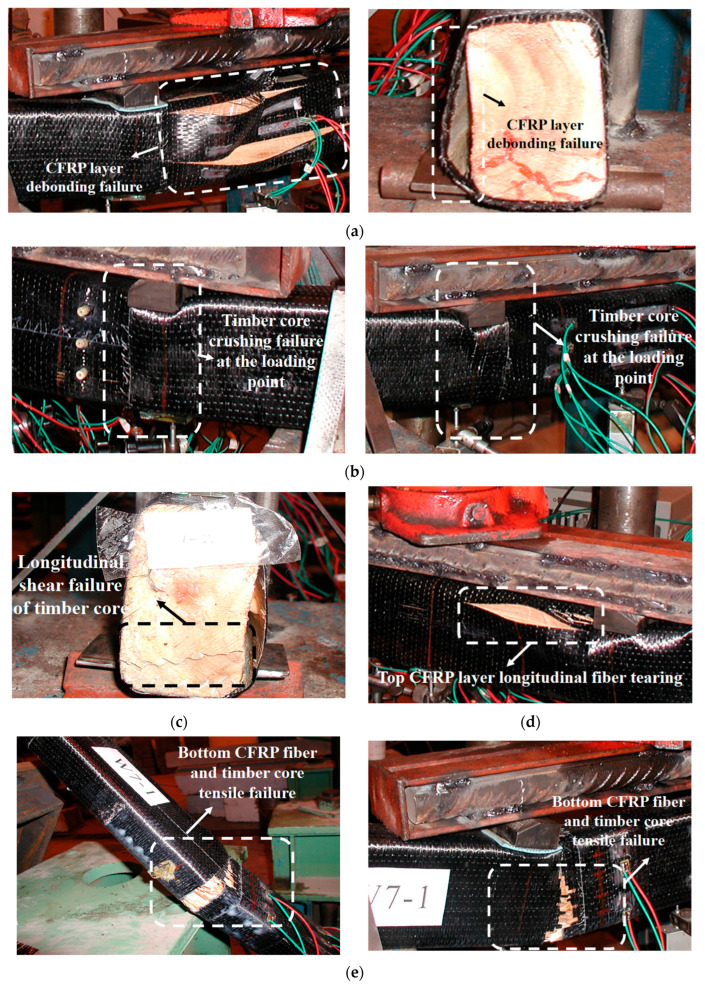

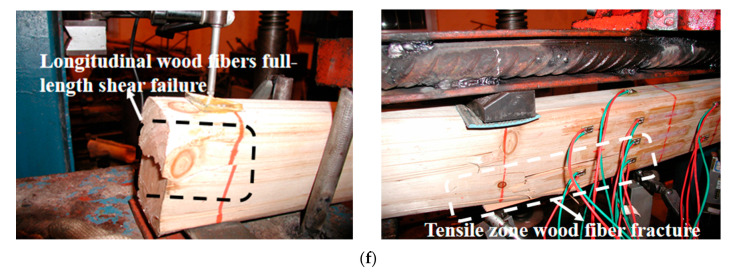
Several typical failure modes of specimens: (**a**) CFRP layer debonding failure, (**b**) timber core crushing failure at the loading point, (**c**) longitudinal shear failure of wood core, (**d**) top CFRP layer longitudinal fiber tearing, (**e**) bottom CFRP fiber and timber core tensile failure, (**f**) longitudinal wood fibers full-length shear failure and tensile zone wood fiber fracture of specimens without CFRP wrapping.

The failure behavior of bonded CFRP–timber hybrid beams was found to be highly sensitive to the longitudinal reinforcement ratio and the presence of circumferential confinement. Based on the observation of 14 four-point bending tests, three distinct failure modes were identified, each governed by different physical mechanisms and exhibiting characteristic morphological features (Figure 5).

CFRP tensile rupture represents the intended failure mode, in which the tensile capacity of the carbon fibers is fully mobilized. This mode was predominantly observed in specimens with circumferential wrapping (W1-P30, W1-B1T1-P50) and those with two longitudinal CFRP layers (W2). Failure was instantaneous and accompanied by a sharp, explosive acoustic emission. Multiple fiber tows fractured simultaneously across the width of the tension face within the pure bending region, typically at or near mid-span. Post-failure inspection revealed that the timber core had undergone a clean, brittle tensile split, propagating vertically from the tension face toward the neutral axis. The rupture plane of the CFRP was oriented perpendicular to the beam axis, consistent with unidirectional fiber failure under uniaxial tension. This failure mode is considered desirable because it indicates that the composite action was maintained throughout loading and that the high strength of the CFRP was effectively utilized.

Longitudinal timber splitting occurred predominantly in unwrapped specimens with one or two longitudinal CFRP layers (W1, W1-B1, W1-B1T1, W2). This mode is fundamentally a shear failure of the timber core, driven by the high interfacial shear stresses that arise from the pronounced stiffness mismatch between the CFRP laminate (210 GPa) and the timber (9.0 GPa). In the absence of transverse confinement, these shear stresses concentrate near the bond line and, upon reaching the rolling shear strength of the wood, initiate a Mode II crack that propagates rapidly along the grain direction. The cracking was often audible as a sustained tearing sound and extended through the entire length of the beam, in some cases splitting the timber core into two separate halves. The CFRP laminate on one side of the beam typically became completely detached following the split. This failure mode is particularly brittle and offers little to no visual warning prior to catastrophic failure.

Localized timber crushing and compressive instability governed the failure of specimens with three longitudinal CFRP layers (W3), where the axial stiffness of the tensile reinforcement substantially exceeded the compressive capacity of the timber core. Visible crushing of timber fibers was first observed at the compression zone beneath the loading points or at the mid-span top face. As crushing progressed, the timber core lost its ability to provide continuous support to the CFRP shell, leading to local buckling or delamination of the CFRP laminate in the compression region. Final failure occurred when the buckled fibers fractured or the core disintegrated. This mode represents a core-limited condition in which the compressive strength of the timber becomes the controlling parameter; further increase in CFRP content beyond this point yields diminishing returns and may even reduce structural efficiency.

Collectively, these observations demonstrate that failure mode can be deliberately steered through appropriate reinforcement design. Circumferential wrapping effectively suppresses longitudinal splitting by providing lateral confinement and shear transfer, thereby promoting CFRP rupture. An optimal reinforcement window exists in which the CFRP content is sufficient to achieve substantial strengthening without overwhelming the core. For the specific timber species and cross-section used herein, this optimum corresponds to two longitudinal CFRP layers combined with circumferential wrapping.

## 4. Load–Displacement Curves

**Figure 6:**
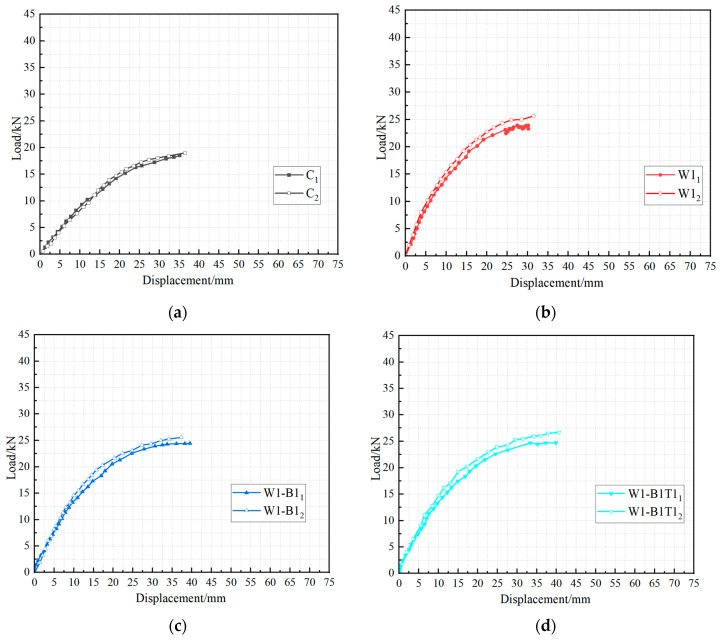

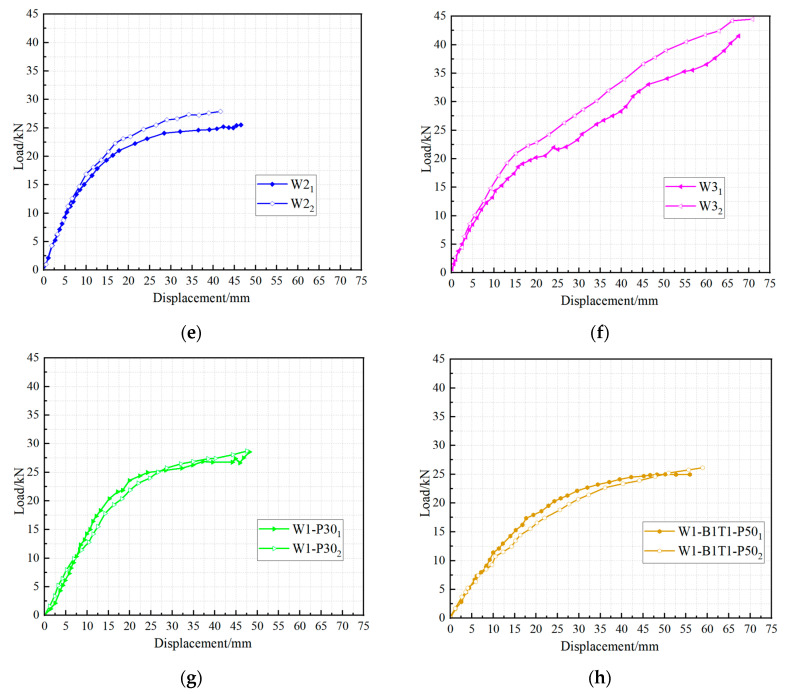
Load–displacement curves for specimens: (**a**) C_1–2_, (**b**) W1_1–2_, (**c**) W1-B1_1–2_, (**d**) W1-B1T1_1–2_, (**e**) W2_1–2_, (**f**) W3_1–2_, (**g**) W1-P30_1–2_, (**h**) W1-B1T1-P50_1–2_.

**Figure 7:**
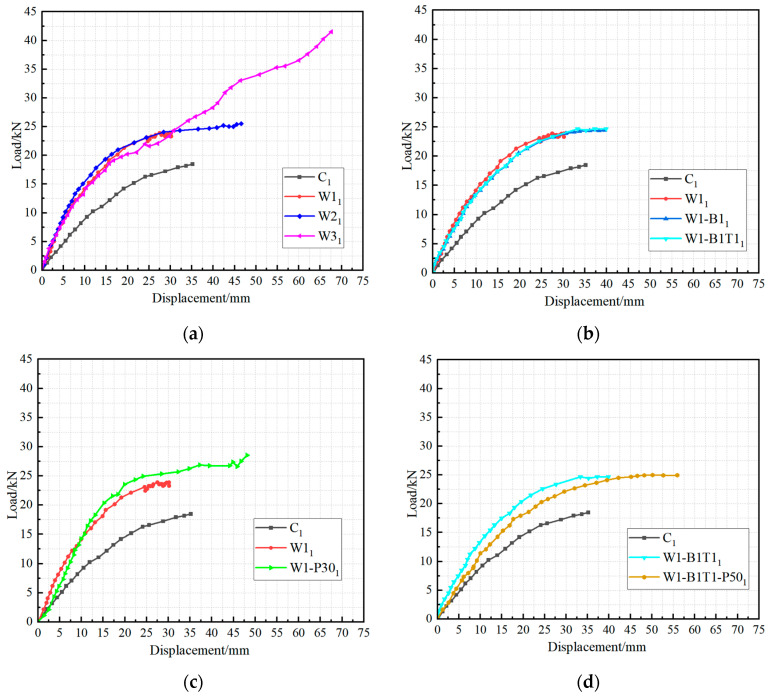
Load–displacement curves for specimens: (**a**) effect of CFRP layer, (**b**) effect of top/bottom CFRP reinforcement, (**c**) effect of lateral reinforcement on pure bending sections (without top/bottom reinforcement), (**d**) effect of lateral reinforcement on pure bending sections (with top/bottom reinforcement).

The load versus mid-span deflection curves for all bonded specimens are presented in Figure 6, with comparative analyses of key parameters shown in Figure 7; the corresponding peak loads and displacements are summarized in Table 2. The response can be divided into two distinct phases. The first phase, from loading onset to the first notable stiffness reduction, exhibits linear-elastic behavior as the beam acts as a fully composite section with uncracked timber and CFRP. This phase ends when the tensile stress at the extreme timber fiber reaches its stress of rupture, initiating micro-cracking and marking a distinct “kink” in the curve. In the second phase, the tensile force previously carried by the timber is abruptly transferred to the CFRP laminate, causing the flexural stiffness to reduce. As loading continues, progressive plasticization (crushing) of wood fibers in the compression zone introduces gradual nonlinearity in the response. This compressive plasticization causes the neutral axis to shift progressively downward toward the tension zone. This phase continues until brittle failure occurs via one of the three mechanisms described previously, characterized by a sudden drop in load capacity with no discernible plastic plateau, reflecting the linear-elastic behavior of the CFRP up to rupture.

The elastic flexural stiffness of the control specimens was 0.84 kN/mm (C); after CFRP reinforcement, the stiffness of the strengthened specimens increased to a range of 1.08–1.85 kN/mm (with an average of approximately 1.5 kN/mm), indicating a significant overall enhancement. However, varying the number of wrapping layers, applying CFRP to the top and bottom surfaces, or using circumferential wrapping had a relatively limited effect on further improving the flexural stiffness.

Increasing the number of longitudinal CFRP layers produced a substantial enhancement in ultimate load. As shown in Figure 7a, the average peak load increased from 24.8 kN for single-layer specimens (W1) to 26.7 kN for two-layer specimens (W2) and further to 42.9 kN for three-layer specimens (W3). The corresponding mid-span displacements at failure also increased markedly, from approximately 31 mm for W1 to 44 mm for W2 and 69 mm for W3. However, this increase in capacity was accompanied by a shift in failure mode from CFRP rupture or mixed splitting (W1, W2) to timber crushing (W3), indicating that the compressive strength of the core becomes the limiting factor beyond a certain reinforcement ratio. The two-layer configuration thus represents a favorable compromise between strength enhancement and efficient material utilization.

The beneficial effect of circumferential wrapping is clearly demonstrated in Figure 7c,d. For specimens with identical longitudinal reinforcement (single layer), the addition of transverse wraps increased the average peak load from 24.8 kN (W1) to 28.7 kN (W1-P30), an improvement of approximately 15%. Moreover, the wrapped specimens exhibited a more stable post-cracking stiffness and consistently failed by the desired CFRP tensile rupture, whereas their unwrapped counterparts were prone to longitudinal splitting. A similar trend was observed for specimens with top and bottom reinforcement: the wrapped series (W1-B1T1-P50) achieved an average peak load of 25.5 kN, while the unwrapped series (W1-B1T1) reached 25.7 kN; the wrapped specimens also exhibited a cleaner failure mode. These results confirm that circumferential wrapping not only increases ultimate capacity but also enhances the predictability and reliability of the structural response.

The addition of extra CFRP strips on the tension face alone (W1-B1) provided only a modest gain in capacity (25.0 kN versus 24.8 kN for W1), and the simultaneous addition of both top and bottom strips (W1-B1T1) yielded no further improvement. This suggests that, for the beam geometry and reinforcement levels considered, the primary contribution of the CFRP derives from the full-width longitudinal layers rather than from localized face reinforcement.

## 5. Load–Strain Curves

**Figure 8:**
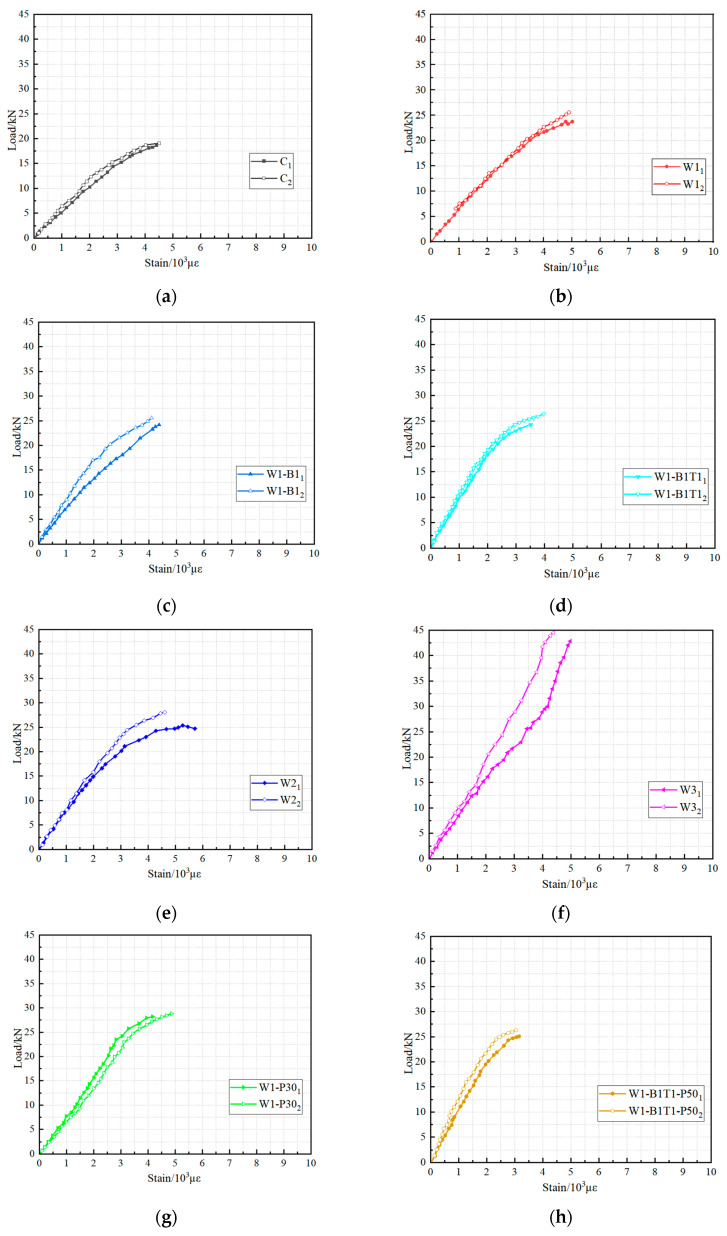
Load–strain curves: (**a**) C_1–2_, (**b**) W1_1–2_, (**c**) W1-B1_1–2_, (**d**) W1-B1T1_1–2_, (**e**) W2_1–2_, (**f**) W3_1–2_, (**g**) W1-P30_1–2_, (**h**) W1-B1T1-P50_1–2_.

The tensile strain developed in the bottom CFRP laminate at mid-span was monitored throughout each test to investigate the stress transfer mechanism and the utilization efficiency of the carbon fibers. Figure 8 presents the load versus tensile strain curves for all bonded hybrid beams; each curve represents the response of a single specimen, with the strain measured on the outermost longitudinal layer at the tension face.

All load–strain curves exhibit a clear bilinear form that correlates closely with the load–deflection response. In the initial stage, before the onset of timber cracking, the tensile strain increases linearly with load, reflecting the fully composite behavior of the uncracked section. The slope of this stage is governed by the combined stiffness of the timber core and the CFRP laminate. A distinct change in slope—a reduction in stiffness—occurs at the load corresponding to the “kink” observed in the load–deflection curve. This transition marks the cracking of the timber tension zone; thereafter, the tensile force is progressively transferred from the wood to the CFRP, and the strain in the CFRP increases at a faster rate. The post-cracking branch remains essentially linear until failure, consistent with the linear-elastic behavior of the carbon fibers.

The magnitude of tensile strain at ultimate load provides a direct measure of how effectively the CFRP material is utilized. For specimens that failed by the desired CFRP tensile rupture (W2, W1-P30, W1-B1T1-P50), the ultimate tensile strain ranged from 9500 to 12,500 microstrain, corresponding to 68–89% of the nominal ultimate strain capacity of the carbon fiber (14,000 microstrain). This high level of strain utilization confirms that the bonded interface was capable of transferring the required shear stresses without premature debonding, and that the circumferential wrapping successfully suppressed longitudinal splitting, allowing the CFRP to approach its full strength.

A clear influence of the longitudinal CFRP content on strain development can be observed. Specimens with two longitudinal layers (W2) attained ultimate tensile strains near the upper end of the range (approximately 12,000 microstrain), whereas single-layer specimens that failed by rupture (W1-P30, W1-B1T1-P50) typically reached slightly lower values. Three-layer specimens (W3), which failed by timber crushing rather than CFRP rupture, exhibited substantially lower ultimate tensile strains (typically below 8000 microstrain), indicating that the CFRP was underutilized due to premature core failure.

The effect of circumferential wrapping is evident when comparing the load–strain curves of wrapped and unwrapped specimens with identical longitudinal reinforcement. At any given load level, wrapped specimens consistently exhibited lower tensile strain than their unwrapped counterparts. For example, at a load of 20 kN, the tensile strain in the wrapped beam W1-P30-1 was approximately 4500 microstrain, while the unwrapped beam W1-1 recorded approximately 5200 microstrain. This behavior is attributed to the confinement provided by the circumferential wraps, which suppresses interfacial slip and micro-delamination, thereby maintaining a more efficient composite section. As a result, the tensile strain demand on the CFRP for a given bending moment is reduced, enabling wrapped beams to sustain higher ultimate loads despite lower strain accumulation rates.

The addition of extra CFRP strips on the tension or compression faces (W1-B1, W1-B1T1, W1-B1T1-P50) did not produce a consistent or significant alteration of the load–strain response beyond that attributable to the changes in longitudinal reinforcement and wrapping. This observation reinforces the conclusion that the full-width longitudinal layers are the primary determinant of flexural performance. Collectively, the tensile strain measurements provide essential experimental evidence for understanding the development of composite action and for validating the efficiency of different reinforcement configurations.

## 6. Conclusions

This experimental investigation into the flexural behaviour of fully bonded CFRP–timber hybrid beams has led to the following principal conclusions:

1. The proposed hybrid beam system is structurally effective and can be fabricated reliably using a carefully controlled hand lay-up process. When a full bond is achieved between the CFRP skin and the timber core, the two materials act compositely, and the beam exhibits substantial enhancements in both stiffness and flexural strength relative to an equivalent solid timber beam. The ultimate moment capacity of the hybrid beams tested in this study ranged from 1.3 to 3.4 times that of the unreinforced controls, confirming the efficiency of the concept.

2. Three distinct failure modes were observed, and each was found to be strongly correlated with the reinforcement design. CFRP tensile rupture, the intended mode, occurred in specimens that were provided with circumferential wrapping in the pure bending region and that contained one or two longitudinal CFRP layers. Longitudinal splitting of the timber core dominated in unwrapped specimens with one or two layers; this mode is driven by high interfacial shear stresses and is undesirable because it prevents full utilisation of the CFRP. Localised crushing of the timber core, accompanied by instability of the compression-side CFRP, governed the failure of specimens with three longitudinal layers, indicating that the compressive strength of the timber had become the limiting resource.

3. Circumferential CFRP wrapping emerged as a critical design detail. Its presence increased the ultimate load capacity by 15–20% for otherwise identical beams, and it consistently shifted the failure mode from brittle splitting to the more predictable and desirable CFRP rupture. The wraps act as both shear reinforcement and confining devices, mitigating the interfacial shear stresses that initiate splitting and enhancing the apparent compressive strength of the core.

4. An optimal longitudinal reinforcement ratio exists for this material system. The two-layer configuration (W2) offered the most favourable balance between strength gain and material economy; the three-layer configuration (W3) produced a further increase in capacity but at the cost of a change in failure mode and a reduction in the efficiency of CFRP utilisation. This finding has direct implications for design: indiscriminate addition of CFRP beyond a certain point yields diminishing returns and may compromise structural reliability.

5. Throughout the test programme, the hybrid beams displayed pronounced linear-elastic behaviour up to the point of sudden brittle failure. No yielding plateau or other warning signs preceded failure. Moreover, a non-negligible scatter in performance was observed, even among nominally identical specimens. This variability is inherent in systems that combine a natural material (wood) with a manual fabrication process, and it underscores the necessity of employing appropriate characteristic material properties and partial safety factors in design, as well as implementing rigorous quality control during fabrication.

The experimental data reported herein provide a comprehensive and reliable basis for the development of analytical and numerical models for the design of bonded CFRP–timber hybrid beams. These data can be used to calibrate numerical simulations and to validate theoretical predictions of flexural behaviour. In fact, preliminary finite element analyses were conducted to benchmark the flexural response of the tested configurations. However, due to the lack of experimentally derived bond–slip data for the CFRP–timber interface at this stage, the initial numerical results exhibited some discrepancies when compared with the experimental measurements. This limitation highlights the critical need for further investigation into interfacial behavior. To address this, future work should focus on the development of a refined finite element model that accurately simulates the interfacial slip behavior and stress transfer, as well as a theoretical model for predicting the flexural capacity. These numerical and theoretical investigations will complement the experimental findings and facilitate parametric studies beyond the tested configurations, with particular emphasis on characterizing the bond–slip relationship as a key priority.

## Figures and Tables

**Table 1:** Parameters of the specimens.

Specimens	Number of CFRP Laying Layers					
	Wrapped on All Sides	Bottom Layers	Top Layers	Transverse Wrapping for Flexural Shear Zones	Transverse Wrapping for Pure Bending Zone	
					Wrapping Width /mm	Spacing Distance /mm
W1_1–2_	1	0	0	1	/	/
W1-B1_1–2_	1	1	0	1	/	/
W1-B1T1_1–2_	1	1	1	1	/	/
W2_1–2_	2	0	0	1	/	/
W3_1–2_	3	0	0	1	/	/
W1-P30_1–2_	1	0	0	1	50	30
W1-B1T1-P50_1–2_	1	1	1	1	50	50
C_1–2_	0	0	0	0	0	0

Note: _1–2_ indicates that two replicate experiments were conducted for each type of test specimen.

**Table 2:** Test results of specimens.

Specimens	Peak Load /kN			Elastic Flexural Stiffness kN/mm			Ultimate Displacement /mm		
	Sample 1	Sample 2	Average Value	Sample 1	Sample 2	Average Value	Sample 1	Sample 2	Average Value
W1_1–2_	23.9	25.6	24.8	1.65	1.77	1.71	30.2	31.5	30.9
W1-B1_1–2_	24.4	25.5	25.0	1.46	1.57	1.51	39.6	37.3	38.5
W1-B1T1_1–2_	24.7	26.7	25.7	1.48	1.64	1.56	39.9	40.6	40.3
W2_1–2_	25.5	27.9	26.7	1.73	1.97	1.85	46.5	41.7	44.1
W3_1–2_	41.2	44.5	42.9	1.50	1.54	1.52	67.6	70.9	69.3
W1-P30_1–2_	28.6	28.7	28.7	1.41	1.23	1.32	48.1	47.5	47.8
W1-B1T1-P50_1–2_	24.9	26.1	25.5	1.14	1.02	1.08	55.9	58.9	57.4
C_1–2_	18.6	19.0	18.8	0.86	0.82	0.84	35.2	36.4	35.8

## Data Availability

The original contributions presented in this study are included in the article. Further inquiries can be directed to the corresponding author.
